# In-situ, time resolved monitoring of uranium in BFS:OPC grout. Part 1: Corrosion in water vapour

**DOI:** 10.1038/s41598-017-08601-x

**Published:** 2017-08-11

**Authors:** C. A. Stitt, C. Paraskevoulakos, A. Banos, N. J. Harker, K. R. Hallam, A. Davenport, S. Street, T. B. Scott

**Affiliations:** 1 Interface Analysis Centre, H. H. Wills Physics Laboratory, University of Bristol, Bristol, UK; 2 European Synchrotron Radiation Facility, Grenoble, Rhône-Alpes France; 3 School of Metallurgy and Materials, University of Birmingham, Edgbaston, Birmingham UK; 4 Department of Materials, Imperial College London, Royal School of Mines, Exhibition Road, London, SW7 2AZ UK

## Abstract

Uranium encapsulated in grout was exposed to water vapour for extended periods of time. Through synchrotron x-ray powder diffraction and tomography measurements, uranium dioxide was determined the dominant corrosion product over a 50-week time period. The oxide growth rate initiated rapidly, with rates comparable to the U + H_2_O reaction. Over time, the reaction rate decreased and eventually plateaued to a rate similar to the U + H_2_O + O_2_ reaction. This behaviour was not attributed to oxygen ingress, but instead the decreasing permeability of the grout, limiting oxidising species access to the metal surface.

## Introduction

In the UK, Intermediate Level nuclear Waste (ILW) canisters contain primarily uranium, aluminium and Magnox alloy swarf encapsulated in a high alkaline grout (pH 10–13) within a stainless steel container. This grout is commonly composed of Blast Furnace Slag (BFS) and Ordinary Portland Cement (OPC) in a 3:1 ratio with 0.4 w/c^1^. The container is then capped with a secondary layer of grout and a mild steel lid, with an air vent to allow gas exchange during grout curing and corrosion of metals. The uranium metal surface may be pre-corroded, exhibiting a thick oxide layer, or, if it is grouted immediately after de-canning from the Magnox cladding, it may retain a fresh metallic surface with limited oxide development. These containers are in dry storage at Sellafield for up to 30 years, where the temperature is generally regulated. Due to Sellafield’s location, the air has a high humidity and contains salt; however, the accessibility of this atmospheric water to the metals within the grout is unknown.

Uranium has a high affinity for oxidising species. Immediately upon exposure to air at room temperature, a thin layer of hyper stoichiometric UO_2+x_ will form, and with prolonged exposure to oxygen, the layer will gradually thicken to form higher oxides (x = 0.06–1) such as U_3_O_8_ and UO_3_^2–5^. It is well established that because uranium has a comparatively large atomic size to oxygen, its lattice diffusion within the oxide is finite^6^. Consequently, oxygen ions are recognised as the mobile species and new oxide develops at the oxide-metal interface^3, 7^. Since oxygen ions must diffuse through the existing oxide to reach the metal, the progressively thicker oxide layer becomes an effective barrier and as such, will significantly slow uranium oxidation rates in air. In comparison, uranium oxidation in water or water vapour is observed to be distinctly faster, producing an oxide of higher porosity and friability as well as a stoichiometry closer to pure UO_2_^3, 8–12^. The basic equations with the respective activation energies for each scenario are^3, 12–14^:

1 $$U+{O}_{2}\to U{O}_{2}\quad \quad {{\rm{E}}}_{{\rm{a}}}=67\mbox{--}77{\rm{kJ}}{{\rm{.mol}}}^{-{\rm{1}}}$$

2 $$U+{O}_{2}+2{H}_{2}O\to U{O}_{2}+2{H}_{2}O\quad \quad {{\rm{E}}}_{{\rm{a}}}=76\mbox{--}{\rm{100kJ}}.{{\rm{mol}}}^{-{\rm{1}}}$$

3 $$U+2{H}_{2}O\to U{O}_{2}+2{H}_{2}\quad \quad {{\rm{E}}}_{{\rm{a}}}=41.8\mbox{--}64.4{\rm{kJ}}{{\rm{.mol}}}^{-{\rm{1}}}$$

In the context of nuclear waste storage^11, 12, 15^, Equation 3 is of most interest as (1) it is the fastest oxidation reaction^3, 11, 12^, quickly converting the unstable uranium metal into a stabilised material. (2) It has the potential to form uranium hydride (UH_3_) at the metal-oxide interface^16–20^. Finally, (3) it releases hydrogen gas that may become trapped within the grout and later react with the uranium to form uranium hydride via Equation 4 ^21^.

4 $$2U+3{H}_{2}\to 2U{H}_{3}\quad \quad {{\rm{E}}}_{{\rm{a}}}=57{\rm{kJ}}{{\rm{.mol}}}^{-{\rm{1}}}$$

Uranium hydride is particularly undesirable since evidence suggests it is a pyrophoric powder^22^. In addition, the volume expansion associated with both uranium oxide and hydride formation may be sufficient to cause grout fracturing and deformation, or rupturing of the container walls, posing a risk to workers and the environment during storage and transport. However, establishing a risk assessment and quantitative analysis of metal corrosion hidden within grout and stainless steel poses a significant challenge.

Current existing literature of uranium corrosion has predominantly been performed on unconfined, bare uranium metal^3, 5, 8, 11, 18, 23–27^ and few studies have investigated the oxidation behaviour of uranium in grout. The most notable are a Serco report^28^ summarising a number of grout-uranium studies, most of which are unavailable in the open literature, and Wellman^29^, who focuses on the interaction of uranium with grout matrices, specifically determining the solubility limiting phases of uranium in aqueous grout equilibrated conditions. In the Serco report Godfrey^30^ studied the oxidation of uranium in BFS-OPC grout and concluded that uranium-grout oxidation corroded at a rate similar to Equation. 3 and that the reaction rate was linear^28^. This latter observation was also supported by Hayes^28, 31^. In contrast, other authors have shown that slow diffusivity of oxidising species from the air through the grout limits metal oxidation^28, 32, 33^; BFS was ultimately chosen for its low permeability qualities^34^. However, a model created by Serco to assess the potential for gas generation within an ILW container called SMOGG (Simplified Model of Gas Generation) assumed periods of oxidising conditions within the waste container during transport and storage, suggesting that gas and water vapour diffusion occurs freely through the vented container and grout^28^. In conclusion, the exact chemical and physical conditions within ILW grout are debatable and hard to distinguish since hydration will alter the grout chemical composition and physical properties over time. Furthermore, little research has been performed on uranium corrosion in anoxic grout conditions despite that BFS is known to form chemically reducing conditions^35^.

The aim of our study was to examine the corrosion behaviour of as-received (pre-corroded) and nitric acid etched uranium metal encapsulated in BFS:OPC grout when exposed to water vapour over a 50 week time period. These conditions were chosen to reflect the environmental conditions found in dry interim storage, and the results provide important information such as the dominant types and rates of uranium corrosion which could be used for predictive corrosion modelling of ILW containers. Synchrotron x-ray powder diffraction (XRPD) and tomography (XRT) were used to analyse the uranium encapsulated in grout, *in situ*.

## Results

In total, 8 samples of uranium metal (rods measuring 0.5 mm × 0.5 mm × 20 mm) were encapsulated in grout and exposed to water vapour for a specific period of time (1 to 50 weeks). All samples were examined once or twice over two sessions at the Diamond Light Source (DLS), on the I12 Joint Engineering, Environment and Processing beamline (JEEP). The samples were split into two groups, described in Table 1. Before encapsulation in grout, three samples retained an as-received corrosion layer on the metal surface thereby reflecting uranium fuel which is pre-corroded before waste packaging. Five further samples represented recently de-canned uranium fuel, and were pre-treated with nitric acid prior to grout encapsulation. The names of the two sample groups begin with a letter A and N respectively followed by a number which accounts for the number of weeks exposed to water vapour.

**Table 1:** A summary of the 8 uranium metal samples including the sample name, type of metal surface pre-treatment, water vapour exposure length and the beam time examined.

Sample name	Pre-treatment	Exposure length (weeks)	Beam time examined
A1	As-received	1	1
A3		3	1
A6		6	1
A47*		47	2
A50*		50	2
N1	Nitric acid etched	1	2
N2		2	2
N6		6	2
N12		12	2
N22		22	2

The sample names preceding with an A (Group A) received no previous surface preparation so were considered the as-received samples. Sample names preceding with an N (Group N) received nitric acid etching prior to grout encapsulation. *The two samples A3 and A6 were re-analysed on the second beam time after further exposure to water vapour. These were then renamed A47 and A50 respectively.

**Figure 1:**
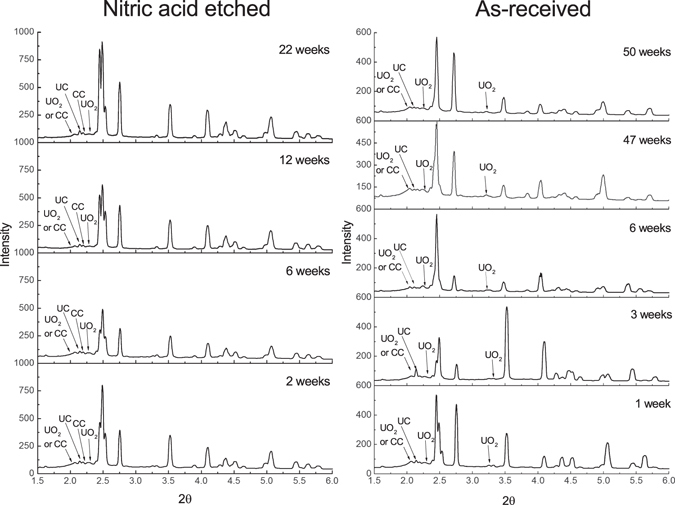
XRPD exhibiting the evolution of nitric acid etched and as-received, grout encapsulated uranium when exposed to de-ionised water vapour over time. CC = Calcium carbonate (CaCO_3_). All unlabelled peaks are attributed to uranium metal.

On each sample XRPD line scan data were averaged and are displayed in Fig. 1. The intensity of the measured corrosion product peaks between samples were not appropriate to compare since the photon flux and extent of attenuation varied between beam times and due to the imperfect geometry of each sample. UO_2_ was identified as the dominant corrosion product in all samples, and no UH_3_ was detected over the 50-week period.

**Figure 2:**
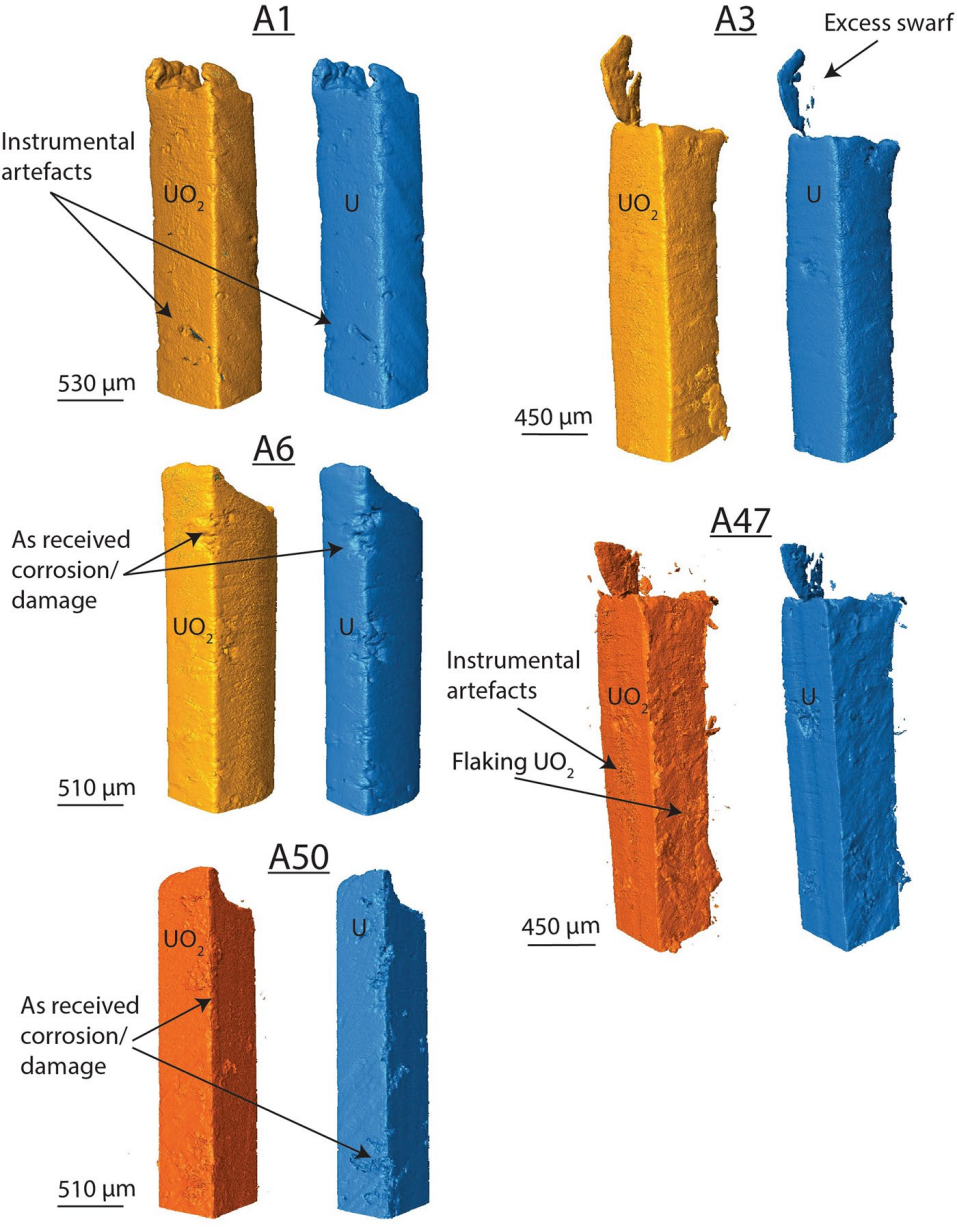
3D renders of the as-received samples after exposure to water vapour. The contrast in density between uranium and UO_2_ permitted rendering of the two materials separately, thus the 3D renders of each sample are displayed in pairs. The images in blue show the residual uranium metal (right) and yellow or orange represents UO_2_ (left). The XRT quality between the two beam times changed dramatically and to show this, 3D renders in yellow are from using higher energies (115.6 keV) and orange from the lower energy (113.3 keV). Generally, edge artefacts, instrumental artefacts and absorption by the uranium prevented clean and sharp 3D renders for the higher energy beam time.

**Figure 3:**
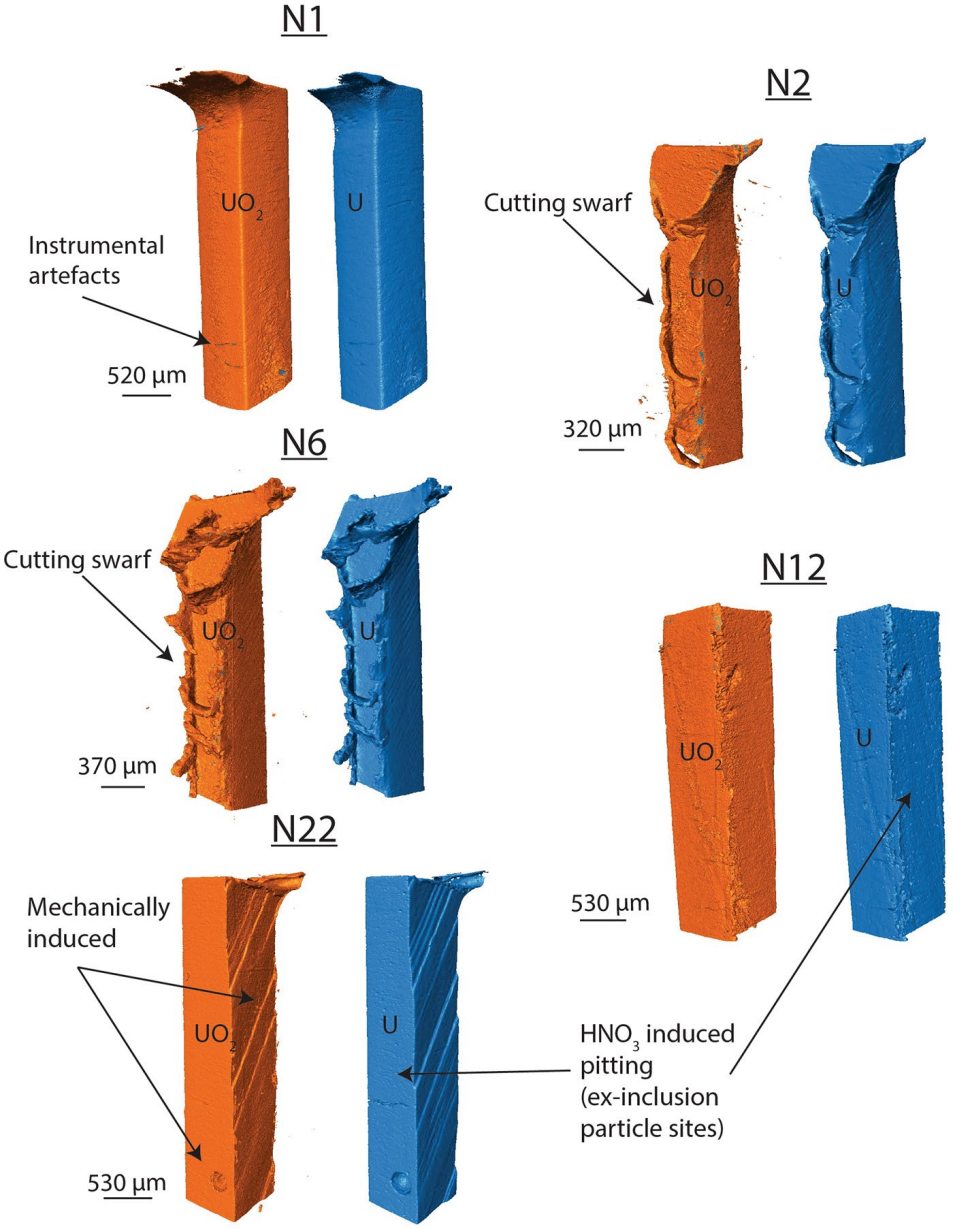
3D renders of the nitric acid etched samples. The corrosion products are exhibited in orange (left) and the uranium in blue (right).

XRT images of each sample are displayed in Figs 2 and 3. As we expected from uranium characterisation before grout encapsulation (see Supplementary Fig. S1) the ‘as-received’ samples (Fig. 2) displayed a rough surface, with large areas of irregular sized pitting (≤250 µm diameter) which were filled with a corrosion product ≤40 µm thick. The pitted areas were localised, with flat and uniform areas between. Only UO_2_ was detected by XRPD, however previous SIMS analysis also indicated carbon contamination (see Supplementary Fig. S2).

The nitric acid etched samples XRT also showed some as-received features (Fig. 3): excess swarf, from high speed cutting of the sample (for example N2 and N6); large spherical holes and ridges assumed to have formed during the metal casting process, as both were present in the corrosion product and uranium metal render; and small ~18 µm diameter pits, with a spatial density of 39.9 pits.mm^−2^ attributed to the pitting of removed inclusion particles after nitric acid etching (see Supplementary Fig. S3). However, consistent with XRPD analysis, uniform growth of a continuous corrosion layer across the metal surface was observed, indicative of uranium oxide formation.

**Figure 4:**
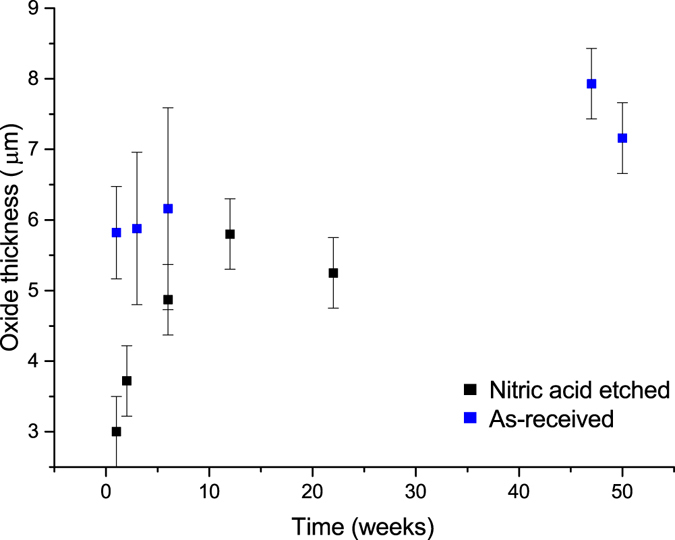
A plot demonstrating the change in UO_2_ thickness on each uranium sample when exposed to water vapour over time. Values and errors are extracted from Table 2.

Overall, we observed limited visual change between grout encapsulated uranium samples exposed to water vapour over progressively longer periods of time. We used cross sections at multiple positions of each sample render to measure the oxide thickness at 80 locations, and the average oxide thickness, with an associated error and range are shown in Table 2. In general, the oxide thickness of the uranium samples was observed to increase over time. More specifically, over the first 12 weeks the nitric acid etched uranium oxide growth appeared to initially grow rapidly, but then the growth rate decreased by 22 weeks (Fig. 4). Oxide thicknesses were greater and displayed a greater range on the ‘as-received’ samples owing to the initial corrosion layer present prior to encapsulation in grout.

**Table 2:** The average UO_2_ thickness observed on each uranium sample.

Sample	Oxide thickness (µm)	Error+/− (µm)	Range (µm)
A1	5.82	0.66	6.79
A3	5.88	1.08	10.54
A6	6.16	1.43	14.59
A47	7.93	0.50	36.16
A50	7.16	0.50	54.97
N1	3.00	1.20	6.19
N2	3.72	0.50	10.91
N6	4.87	0.50	24.46
N12	5.80	0.50	13.82
N22	5.25	0.50	10.02

Measurements were obtained from 80 locations on multiple cross sections of the XRT 3D renders. Errors in measurements were caused by X-ray edge artefacts, micro-porosity in the UO_2_ and minor overlapping of phase densities between the grout and UO_2_. Error estimates were obtained by measuring the oxide thickness three times: at the lowest, middle and highest threshold x-ray signal perceived to represent the UO_2_ for rendering the 3D image. The middle measurement was then used as the final value, with the associated maximum and minimum measurement value error range. The range column represents the range in thicknesses across the surfaces of each sample, which varied according to the geometry of the sample surface. For example, the corners of each rectangular sample generally exhibited greater oxide thicknesses than the flat face surfaces.

## Discussion

Our experiment examined the corrosion behaviour of as-received and nitric acid etched uranium metal encapsulated in grout and exposed to water vapour over time. We used synchrotron XRPD and XRT to identify the arising uranium corrosion products and for morphological analysis respectively. In all instances, each sample showed a degree of UO_2_ growth on the metal surface and no evidence of uranium hydride growth over the 50-week period.

**Figure 5:**
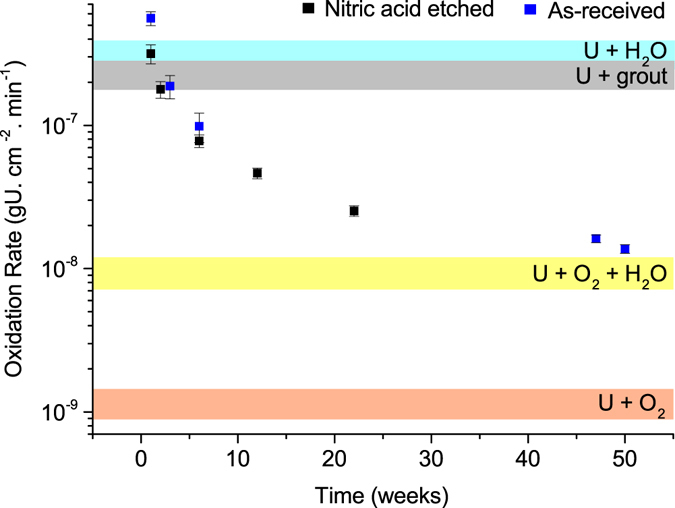
A plot demonstrating the variability in oxide growth rates of grout encapsulated uranium exposed to water vapour over time. Each band of colour represents the oxidation rate at 25 °C calculated from the empirically derived equations displayed at the beginning of this section by^12, 13, 30, 36^. These rates are compared to the rates calculated from the oxide thicknesses determined here. The error bars originate from the errors exhibited in Table 2, which have been processed through the equations.

To indicate the predominant mechanism for corrosion in the grouted system, we compared the rates of oxidation observed here to the empirically derived linear rates from the literature, in Fig. 5. The derived Arrhenius rate expressions used were:

The U + O_2_ reaction for ≤200 °C from Haschke^13^

5 $$K={e}^{6.19-(\frac{8077}{T})}$$

The U + H_2_O + O_2_ reaction for 25–100 °C from Ritchie^12^ and Delegard and Schmidt^36^

6 $$K=\frac{{10}^{(9.466-(\frac{3836}{T}))}}{60000}$$

The U + H_2_O reaction for 10–350 °C from Delegard and Schmidt^36^

7 $$K=\frac{{10}^{(9.9752-(\frac{3564.3}{T}))}}{60000}$$

The corrosion rates of uranium in BFS/OPC grout determined by Godfrey *et al*.^28, 30^.

8 $$K=\frac{3.32\times {10}^{11}\times {e}^{(-\frac{77800}{RT})}}{60000}$$

where the rate K = gU.cm^−2^.min^−1^ and temperature T = 299.15–304.15 K and R = 8.314 J.mol^−1^K^−1^, the universal gas constant. Allowing 2 hrs for the uranium to oxidise in air prior to grout encapsulation permitted the assumption that the uranium oxidation rates in all samples had exceeded the initial fast parabolic stage and proceeded to linear growth^15, 37, 38^. We calculated the average UO_2_ growth rate for each sample from the average UO_2_ thickness obtained from XRT measurements (Table 2) and the time period over which each sample was reacted. For simplicity the oxide growth rate across the metal surface was assumed to be equal.

Figure 5 shows that the oxidation of grout encapsulated uranium, which had been exposed to water vapour for increasingly longer time periods, initially proceeded at rates similar to those observed for the rapid U + H_2_O oxidation regime reported by Delegard and Schmidt (6). However, over time the oxidation rates gradually decreased towards an apparent U + O_2_ + H_2_O regime, with a decay rate (D) of (3.96 × 10^−7^)T^−0:86^, where T = time (weeks) (Fig. 5). It is believed that this behaviour was directly related to the ageing properties of the grout.

Hydration and maturation of OPC and BFS typically involves the development of mineral and C-S-H phases (where C = CaO, S = SiO_2_ and H = H_2_O). Usually, these materials grow around grains and volumetrically reduce the grout pore network over the first 2 months of development^39^. BFS was chosen particularly for its ability to reduce the grout permeability (0.3 × 10^−13^ m.s^−1^ ^40^ in comparison to 1.0 × 10^−13^ m.s^−1^ of pure OPC^41^), as well as its low temperature of hydration and high fluidity which together act to reduce metal oxidation rates, fill all available spaces around the uranium and ultimately reduce gas (O_2_ or H_2_O_(g)_) diffusion pathways to and from the metal surface^1^. Consequently, this was expected to have greatly affected the uranium-grout oxidation system.

In unconfined conditions at temperatures <35 °C, oxygen is believed to compete and dominate over water in the U + H_2_O reaction^11, 42^. However, here, it is anticipated that the physical barrier of the grout and its decreasing permeability over time severely limited further oxygen diffusion from the air through the grout to the metal surface. Thus, initially it is expected that a brief U + O_2_ + H_2_O oxidation regime occurred at the time of grout encapsulation from oxygen trapped within pore waters, but this was quickly followed by the prevailing U + H_2_O oxidation regime during the first 2 weeks of grout curing. Since the grout was still within the early stages of hydration at this time, water was probably still plentiful and available to achieve this. The decreasing uranium oxidation rate from this point could then indicate slow ingress of oxygen into the grouted system over time. However, it is more likely that as the grout hydrated, water was depleted through increasing formation of C-S-H phases thus reducing the grout permeability further. Uranium oxidation was therefore slowed by the decreasing availability of diffusion pathways for other types of oxidising species such as O^2−^ or OH^−^ from H_2_O, toward the metal surface. This is important since many oxidation rate equations assume a constant source of water and a linear rate of corrosion, e.g. Godfrey^28, 30^. Evidence suggests if the grout is intact, that over a 50-week period this is not the case. This behaviour has also been observed in the literature. For example, using electrochemical techniques the corrosion rates of uranium encapsulated in 3:1 PFA/OPC grout revealed a decrease in oxidation rate over time, which was revived upon exposure to fresh distilled water^28, 32, 43^.

Toward 50 weeks, the uranium oxidation rates change again and are observed to plateau (Fig. 5). This may suggest the beginning of a linear rate which is governed by the established permeability of a near fully matured grout, achieving a steady state which allows some slow diffusion of water vapour to the metal surface.

## Conclusion

From the gathered data, we conclude that when exposed to water vapour, uranium encapsulated in grout quickly established an anoxic oxidation regime following depletion of residual atmospheric oxygen in the grout pore waters. We observed the oxidation rate to decrease over time, and hypothesise that the supply of oxidising species was limited by the physical structure of the grout. For ILW these results suggest that oxidation is relatively slow but since BFS is known for its reducing environment, hydrogen generation is possible. If diffusion away from the metal surface is slow and closed porosity dominates, over long periods of time hydrogen concentrations may increase at the metal surface. Thus if the conditions are right, there is potential for uranium hydride to form.

## Materials and Methods

### Sample preparation and reaction

The two groups of uranium rods were reacted identically excluding two differences. (1) All uranium described here originated from Magnox Ltd. The uranium rods used for Group ‘A’ (Table 1) were cut from a uranium coupon that was manufactured earlier in the Magnox program than the coupon used for the Group ‘N’ (Table 1). The uranium in the second group was therefore expected to retain higher concentrations of impurities, but this was not expected to significantly alter the corrosion results. (2) The two different pre-treatments described previously. Group A samples were cut from an aged uranium metal coupon using a Struers Accutom and subsequently rinsed and cleaned first with acetone and then high purity methanol in a sonicator for 5 minutes each. The samples were then left in air for 2 hours to ensure that an oxide had formed on the cut metal surface and that all succeeding chemical reactions ensued through this interface. A second synchrotron analysis was also possible for samples A3 and A6 after further exposure to water vapour, the results of which are labelled under A47 and A50 respectively.

After cutting, Group N uranium rods were abraded successively on all surfaces from grades p600–2500, using SiC grit paper and water as lubricant. The uranium rods were then submersed in 5 M HNO_3_ for 3 hrs until they appeared ‘shiny’, before being rinsed and cleaned in acetone and then high purity methanol in a sonicator for 5 minutes each. As before, the samples were left in air for 2 hrs prior to grout encapsulation. Characterisation of these samples prior to grout encapsulation are shown in Supplementary Information.

All samples were subsequently encapsulated in a mixture of BFS:OPC in a 3:1 ratio with 0.4 w/c. The resulting cylindrical specimens (13 mm diameter and 35 mm in length) were cured in their mould for 3 days in a moist environment before transfer to their reaction cells. All corrosion environments were set up in a clean test tube at room temperature for the designated period of time. Within the test tube was 5 ml of de-ionised water and the grouted sample was placed on top of a stainless steel M6 bolt. The top of each test tube was wrapped in parafilm. Before transfer to DLS, each sample was removed from its environment and remounted in a customised, hermetically sealed, quartz glass – stainless steel cell and evacuated to 1 × 10^−5^ mbar overnight.

### Equipment and settings

X-ray Powder Diffraction and X-ray Tomography were performed on each sample separately on the I12 (JEEP) beamline, Diamond Light Source. Two sets of beam time were used to examine all samples. For the first session, energies of 114.6 keV and 115.6 keV were used for XRPD and XRT respectively, however this was reduced to 113.3 keV for both techniques in the second session since this energy was further away from the uranium K absorption edge (115.6 keV) and thus produced sharper XRT results. 2D XRPD data were recorded using a flat panel Pixium RF4343 (Thales) in high resolution mode (2880 × 2881 pixels). This detector has a pixel size of 148 × 148 μm and beam size of ~340 × 340 μm. The high resolution PCO pco.4000 imaging detector with its Module 4 camera was used for imaging using a monochromatic beam to obtain the best resolution, 1 pixel = 0.98 × 0.98 μm. Data Analysis WorkbeNch (DAWN) software^44^ was used to view and reconstruct the XRT images of each sample and Avizo® was used to produce 3D renders of the XRT data using the *generate surface* module for specific ranges in greyscale (X-ray intensity) representing each examined material.

On each sample, two horizontal XRPD line scans were performed at different heights across the 0.5 mm width of the metal. To identify the corrosion phases, beam calibration was required once per beam time using a CeO_2_ calibrant (NIST - Standard Reference Material 674b). Since the uranium rod position varied within the grout for each sample, the sample to detector distance varied by 1–2 mm from the central CeO_2_ position, thus small shifts in the 2θ value of the XRPD peaks were expected when comparing data. DAWN software for 2D diffraction and processing tools^45^ were used to convert data from 2D powder diffraction patterns to 1D.

Data underlying this article can be accessed on Zenodo at https://doi.org/10.5281/zenodo.834900, and used under the Creative Commons Attribution licence.

## Electronic supplementary material

Supplementary information
