# Supplementary material for: In-situ, time resolved monitoring of uranium in BFS:OPC grout. Part 1: Corrosion in water vapour
Source: Sci Rep. 2017 Aug 11;7:7999. doi: 10.1038/s41598-017-08601-x (PMC5554164; doi:10.1038/s41598-017-08601-x)
Supplement: Supplementary file 1 — Supplementary information [file 41598_2017_8601_MOESM1_ESM.pdf]

In-situ, time resolved monitoring of uranium in BFS:OPC grout. Part 1: Corrosion in water vapour.

C. A. Stitt, C. Paraskevoulakos, A. Banos, N. J. Harker, K. R. Hallam, A. Davenport, S. Street, T. B. Scott

Materials and Methods Supplementary Information

As-received samples:

Examples of the surfaces present on the as-received samples from focused ion beam and secondary electron microscopy imaging are displayed in Fig. S1. Magnetic sector secondary ion mass spectrometry, in negative ion mode at 30 kV and 3 nA beam current, was used to analyse the 'as-received' surface. The dominant corrosion products observed were carbon - 14 (C^-) and oxides - 16 (O^-) (Fig. S2). From the images, two types of oxide corrosion product morphology were observed on the as-received metal surface. Firstly, the oxide was present in a thin layer, characterised by coarse grooves and flakey morphology. The second morphology present were isolated islands of thick oxide accumulations, demonstrating previous localised corrosion. Patches of carbon contamination were also present. In reality, uranium contained in ILW drums will feature an initial corrosion layer formed during pond storage and subsequent drying.

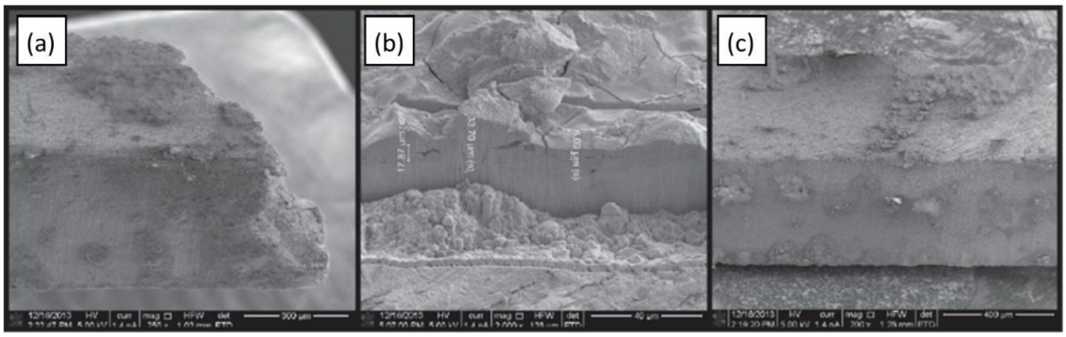

Figure S1. Secondary electron microscopy imaging of the 'as-received' uranium metal surface. An irregular surface is observed with localised areas of thick corrosion product.

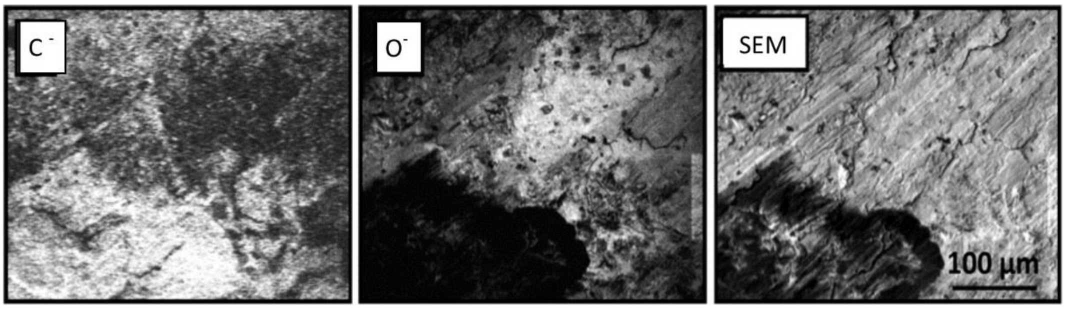

Figure S2. Magnetic sector secondary ion mass spectrometry mass ion maps taken in negative ion mode of the main impurities C^- and O^- present on the 'as-received' uranium metal surface.

Nitric acid etched samples:

Typical of acid etching procedures, Fig. S3 exhibits removal of inclusion particles from the metal surface, resulting in a pitted uranium surface. Some cutting swarf also remained attached to the uranium rods.

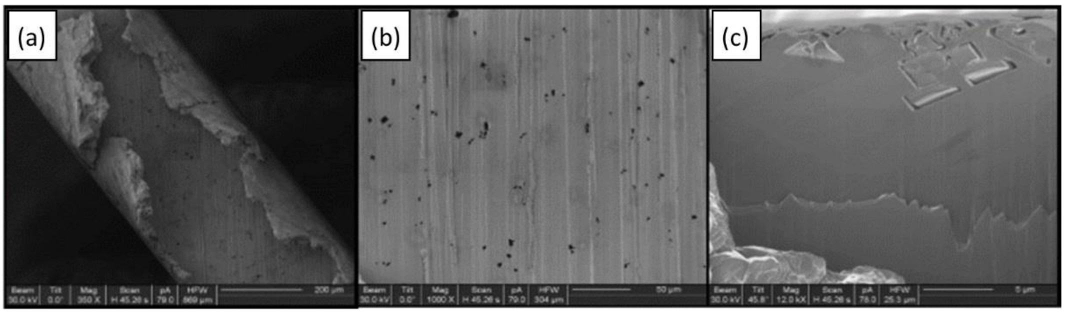

Figure S3. Nitric acid etched uranium surface. Image (a) shows the remaining swarf left over from the cutting procedure. Image (b) demonstrates the intensity of acid etching and exhibits empty pits without inclusion particles. Image (c) shows a cross section of two remaining inclusions at the metal surface and the work hardened layer created from mechanical abrasion.
